# Supplementary figures and images for: Native Aortic Root Thrombosis in Hypoplastic Left Heart Syndrome: An Unusual Presentation (Soon after Atrial Septal Stenting) of a Relatively Unusual Complication—Experience and Literature Review with an Outlook to Diagnosis and Management
Source: J Clin Med. 2023 Aug 17;12(16):5357. doi: 10.3390/jcm12165357 (PMC10455892; doi:10.3390/jcm12165357)

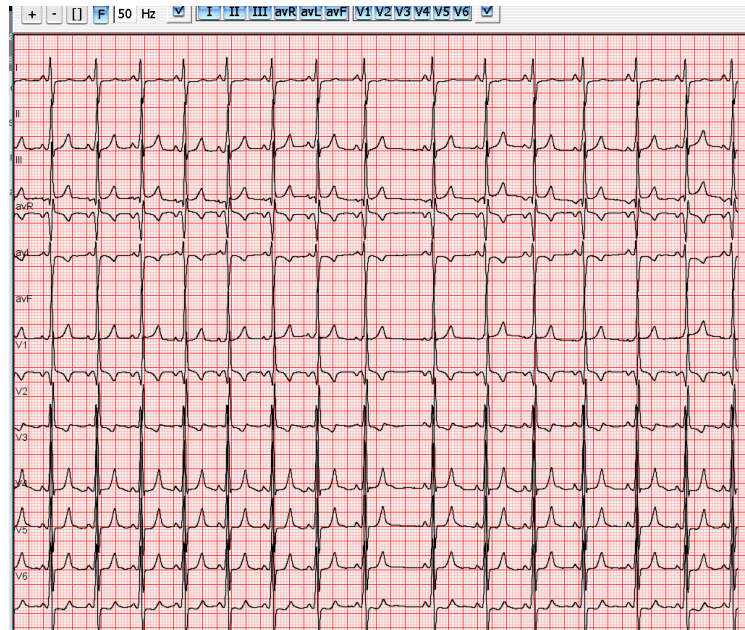

**Supplementary Figure S1.** The ECG showing no significant signs of ischemia.

Supplement: Supplementary file 1 [file jcm-12-05357-s001.zip › Figure S1.pdf]
